# Forceps, Actual Use, and Potential Cesarean Section Prevention: Study in a Selected Mexican Population

**DOI:** 10.1155/2015/489267

**Published:** 2015-08-24

**Authors:** Rodrigo Ayala-Yáñez, Paulette Bayona-Soriano, Arturo Hernández-Jimenez, Alejandra Contreras-Rendón, Paulina Chabat-Manzanera, Roberto Nevarez-Bernal

**Affiliations:** ^1^Obstetrics and Gynecology Division, ABC Medical Center, I.A.P., Avenida Carlos Graef Fdez. 154, Santa Fe, 05300 Mexico City, Mexico; ^2^Colegio Mexicano de Ginecología y Obstetricia (COMEGO), Torre WTC, Montecito 38-29 Of. 21, Col. Nápoles, Benito Juárez, 03810 Mexico City, Mexico

## Abstract

*Objective*. Assessment of the frequency of complications observed with various forceps and operative vaginal delivery (OVD) techniques performed at the ABC Medical Center (Mexico City) to evaluate their safety, bearing in mind the importance of decreasing our country's high cesarean section incidence. *Methods*. We reviewed 5,375 deliveries performed between the years 2007 and 2012, only 146 were delivered by OVD.  *Results*. Only 1.0% of the cases had a serious, life-threatening situation (uterine rupture). The Simpson forceps was the most favored instrument (46%) due to its simplicity of use, effectiveness, and familiarity. Prophylactic use was the most common indication (30.8%) and significant complications observed were vaginal lacerations (*p* = 0.016), relative risk (RR) of 3.4 (95% confidence interval [CI]: 1.15–10.04), and fourth degree perineal tear (*p* = 0.016), RR of 3.4 (95% CI: 1.15–10.04). *Conclusions*. Forceps use and other OVD techniques are a safe alternative to be considered, diminishing C-section incidence and its complications.

## 1. Introduction

Mexican population is estimated to be over 120 million of which 78% lives in urban areas [1, 2]. This implies a significant burden to provide medical care where GDP is nearly $1,300 billion USD and average income per capita is fairly below $10 thousand USD. Yet nearly a fourth of its citizens is deprived of adequate medical services. Among the obstetrical population, this has derived in abuse of cesarean sections (CS) achieving 45.1% of the total births in Mexico. According to the World Health Organization, this figure should be under 15% [3], our private and governmental institutions average 69.6% and 40.6%, respectively [4], well above the WHO recomendation. These ciphers resemble those in regions like Brazil (>30%), Chile (40%), and the United States of America (32.8%) [5, 6].

CS have an increased risk of intraoperative complications (18%), excess blood loss (9%), blood transfusions (1%), febrile morbidity (20%), wound infection (6%), urinary tract infection (6%), neonatal respiratory morbidity (3%), and other critical situations like venous thromboembolism [7–10]. This surgical procedure is effective in saving maternal and infant lives but only when they are required for adequate medical reasons [11].

Vaginal deliveries performed with safe practice of forceps and vacuum extraction techniques may help diminish the increased CS rates. In the USA only 3.4% of the births in 2012 were assisted by forceps or vacuum extraction, compared to 1990 where forceps and vacuum extraction deliveries represented 9.01% of all births [12]. These OVDs can safely assist a vaginal delivery during the second stage of labor (time elapsed from full cervical dilation to neonate delivery, nulliparas beyond 3 hours and multiparas beyond 2 hours) and are associated with lower maternal morbidity without increasing neonatal complications [13].

## 2. Materials and Methods

A total of 5,375 deliveries performed at the ABC Medical Center (from January 2007 to December 2012) were reviewed. In 146 cases forceps was used at delivery, and were evaluated for parameters on maternal forceps indication, type of forceps utilized, gestational age, neonatal birth weight, previous gestations and C-sections, Apgar scores, surgical bleeding, and reported maternal and neonatal complications. All providers are certified obstetricians by Mexican boards and standards, with a minimum 5-year experience as obstetrical practitioners. Statistical analysis was performed using SPSS 20.0. (IBM, Armonk, NY, USA). Approval from the ABC Medical Center Research Committee and Ethics Committee was obtained.

## 3. Results

From a total of 5,375 deliveries selected for this study, 66.3% were C-sections and 33.7% vaginal deliveries with a forceps rate of only 2.55% (included in the vaginal delivery percentage). The average maternal age was 30.9 ± 4.7 years and the average gestational age was 39 ± 1 weeks; 56% were in their first pregnancy and 26% in their second, although we did have a patient with 12 and 9 previous gestations. Most common forceps indications were prophylactic (30.8%), extended expulsion period (30.8%), and persistent posterior-occipital presentation (18.5%) (see Table 1). As for the most favored forceps, Simpson (46.2%), Kielland (32%), and Salas (11%) were the three most frequently used (see Table 2). Specific types of forceps applied for the indications mentioned above are described in Table 1.

Neonatal outcomes had no serious complications, with median Apgar scores at 1 min of 8 and 9 at 5 min. Further findings were 7 (5.5%) abrasions and 2 (1.4%) facial lacerations. Only one neonate was admitted to the neonatal intensive care unit (NICU) due to a gestational age of 34.3 weeks and low Apgar scores (not OVD related). No other complications were reported.

**Figure 1:**
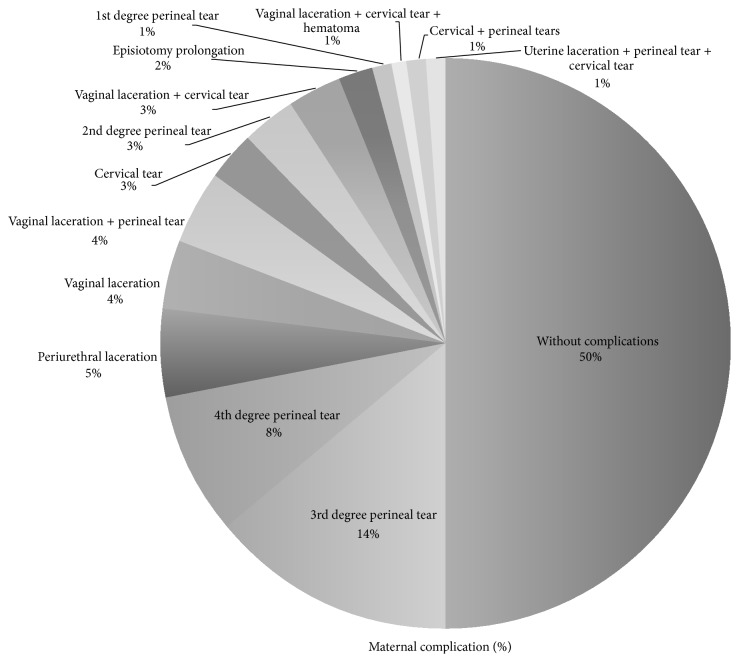
Maternal complication rates found in our study group are low and multiple lesions have been included, none of them were life-threatening except for uterine rupture. Half of our population had no maternal complications reported.

**Figure 2:**
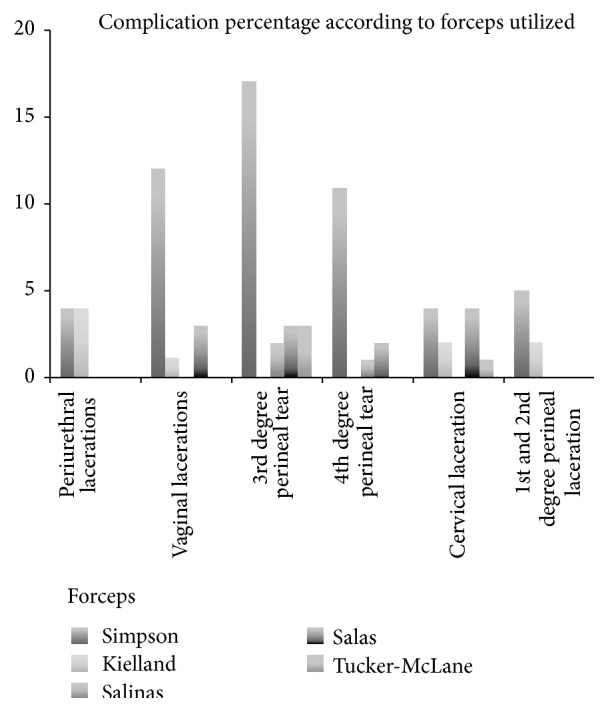
The most common complications found were lacerations to different pelvic structures. Simpson forceps (the most employed instrument) had a significant association with vaginal lacerations (*p* = 0.016), RR of 3.4 (95% CI: 1.15–10.04), OR of 3.93 (95% CI: 1.20–12.84), third degree perineal lacerations (*p* = 0.02), RR of 2.41 (95% CI: 1.12–5.22), OR of 2.89 (95% CI: 1.16–7.22), fourth degree lacerations (*p* = 0.012), RR of 4.16 (95% CI: 1.21–14.28), and OR of 4.77 (95% CI: 1.27–17.95). Salas forceps were significantly associated with cervical tears (*p* = 0.006), RR 4.53 (95% CI: 1.49–13.8), OR 5.71 (CI: 1.46–22.36).

The most common maternal complications associated with the use of forceps were third degree perineal lacerations (17.12%), fourth degree perineal lacerations (8.9%), and cervical lacerations (6.16%) (Figure 1); 50% of the OVDs were free of any maternal complication and no significant association was seen with neonatal birth weight, albeit a third degree laceration was present in the highest birth weight reported (average: 3126 ± 370 g). Surgical hemorrhage was nonsignificant when associated with the type of forceps employed (*p* > 0.05), although fourth degree lacerations utilizing the Simpson forceps had the largest blood loss average volume (480.8 ± 80 mL). Specific complication risk associated with particular types of forceps employed was evaluated, finding vaginal and perineal lacerations as a common complication in Simpson forceps use (*p* < 0.05) and cervical lacerations using Salas forceps (*p* = 0.006) (see Figure 2).

Thirteen cases with prior CS were delivered through forceps application and no significant complications were reported; only 9 had cervical lacerations and 6 had third degree perineal tears. The preferred forceps in these cases was Simpson (46%) with prophylaxis as indication due to the prior CS as risk factor (see Table 2).

## 4. Discussion

The CS surgery was initially introduced as an emergency technique, alternative to vaginal delivery; but increased CS rates are associated with higher postpartum use of antibiotics, severe maternal morbimortality, and higher fetal mortality rates, with an increasing number of neonates admitted to intensive care units [14, 15]. Worldwide, CS have an approximate cost of $2.32 billion US dollars, the estimated cost of actual required CS is $432 million US dollars, and only 54 countries had CS rates under 10%. CS fails to evidence benefits on maternal and neonatal morbidity and mortality rates when their incidence is above 15% [16, 17].

Various factors that influence an increase in C-section rates are as follows: (a) healthcare providers perceiving C-section as a safer procedure over vaginal delivery; (b) limited OVD trained personnel available to attend the on-growing obstetric population; (c) greater number of advanced maternal age cases; (d) increased misinterpretations of cardiotocographic monitoring; (e) social preference for C-sections over vaginal delivery; and (f) economical incentives related to private insurance companies favor C-sections [5]. There is enough evidence to support the notion that nonmedical factors related to obstetricians and pregnant women play an important role in the delivery modality selection [17].

According to Gei et al. [18, 19], the decision to employ a forceps or vacuum should take into consideration three things: (1) increase of expelling force; (2) decrease of resistance of the birth (maternal) channel correcting mal-presentations, asynclitism, and deflections, and (3) decrease of resistance of the birthing channel, increasing the soft pelvis perimeter. These parameters are the basis for the prophylactic use in our study group; most of these cases were extended 2nd labor stage, previous CS history, and maternal exhaustion. Station classification is not used in Mexico, and Hodge planes are preferred due to their anatomical specificity, being that all forceps were applied on 2nd or 3rd Hodge plane [20].

Obstructed labor is a major cause of maternal mortality in developing countries and the WHO has determined that 8% of maternal deaths are due to this complication. Prophylactic use of forceps is quite frequent and is applied to cases including prolonged second stage of labor, suspected fetal jeopardy, and maternal benefits provided by a shortened second stage [21]. Our study supports this observation since prophylaxis was the most common indication, followed by extended expulsion period and persistent posterior-occipital presentation; the suitability of the forceps under these conditions remains to be demonstrated. Few studies have actually evaluated cases using forceps when no assistance was required and determine morbidity rates and attributable risk [19, 21, 22]. Cardiotocography readings are a major factor in deciding for an OVD or a C-section. This is due to poor, inexperienced cardiotocographic interpretations, erroneously diagnosing fetal distress. The fact remains that in most of these cases the preferred procedure is C-section [21]. In our study 2% of the OVDs were indicated due to nonreassuring results, with no complications reported in newborns.

Forceps application may have various neonatal complications, the most serious ones being fetal death, neonatal brain damage, and maternal death [23]. Among the complications associated with forceps we only found 7 cases of abrasions and 2 lacerations; other findings like neonatal ecchymosis, caput succedaneum, cephalohematoma, subarachnoid hemorrhage, brachial plexus lesion, facial paralysis, skull fracture, corneal compression injury, retinal hemorrhage, and subgaleal hematoma were reviewed, yet none of these were found in our study [23, 24]. In order to avoid complications one must try to employ nonoperative interventions to reduce OVD rates although they must be kept in mind prior to moving on to a CS [23].

Risk factors for complications include older women, nulliparous women, heavier fetuses, some variety of dystocia, abnormal contractility patterns, and fetal mal-presentations [19, 21, 25]; many of these factors were absent in this study, since most of our patients were primiparous and the average neonatal weight was 3126 ± 370 g. The few newborns weighing over 3,200 g had no serious complications. Maternal age is always a concern due to chromosomopathies, maternal near miss, preterm deliveries, stillbirths and neonatal complications; still the risks tend to increase in patients after 35 years of age [26]. In our study the average age was 30.9 ± 4.7 years and the one complication was a preterm 34.3-week-old, admitted to the NICU due to low Apgar scores.

Forceps has achieved greater acceptance in our region over vacuum, hence the low incidence and data availability with the latter. Experience recommends Simpson, Salas, and Tucker McLane as prophylactic forceps; our study shows that Kielland (30.8%) was the most selected for this indication, followed by Simpson (26%) (see Table 1); we believe that the main reason here is the experience, familiarity, and preference the physicians at our hospital have with this particular instrument. For prolonged expulsive periods, the favored forceps was Simpson (30.8%), as well as for posterior-occipital presentation (18.5%) (see Figures 1 and 2). Kielland forceps is well known for asynclitism, deflection, and mal-rotation correction, together with other instruments as Salinas and Salas (both designed and published in Mexico) [26]. Each case must be individualized and the physician's experience should be taken into account when selecting the most adequate instrument [27, 28].

Instrumental deliveries are perceived with a high morbidity; misinformation as well as the severity of the worst cases reported have undermined the usefulness of the OVD. Perineal lacerations in various degrees were the most prevalent complications (third and fourth degree) together with cervical lacerations. Although all forceps types were quite safe to use, most complications were associated with the Simpson class, although it was the most employed (46.2%), and the only statistically significant lesion associated with it was vaginal channel lacerations (*p* = 0.016). A significant finding was the Salas forceps association with cervical lacerations (*p* = 0.005), considering its most frequent indication being extended expulsion period (only 18% of the cases) (see Figure 2). We believe that Salas forceps extensive toes and unfenestrated design may have been responsible for these cervical lesions.

We recognize the need of a larger sample to further evaluate the complication rates, proper use, and indications of forceps and other OVDs. This does not attenuate the need to train professionals on forceps and vacuum use. Our hospital is a sample of what happens in our region and would definitively benefit from an increased utilization of forceps and OVD when properly indicated.

## 5. Conclusions

Although a small percentage of forceps applications are performed, complication rates are remarkably low and when present few are severe or life-threatening. Forceps are an underused option prior to CS, which could properly solve various obstetric situations while contributing to diminishing CS increasing incidence rates.

## Figures and Tables

**Table 1:** Forceps indications, their incidence, and the two most selected in each indication.

Indication	Incidence	Selected forceps
Excessive analgesia	0.68%	(1) Simpson

Ominous cardiotocographic tracing	2.00%	(1) Kielland, (2) Simpson

Meconium	2.05%	(1) Simpson, (2) Kielland

Asinclitism	6.16%	(1) Simpson, (2) Kielland

Persistent transverse presentation	6.85%	(1) Simpson, (2) Kielland

Persistent occipital-posterior presentation	18.50%	(1) Simpson, (2) Kielland (equally selected)

Extended expulsion period	30.80%	(1) Simpson, (2) Salas

Prophylactic forceps	30.80%	(1) Kielland, (2) Simpson

This table shows the ascending incidence rates and most favored forceps used for each specific indication. In the persistent occipital-posterior presentation, both forceps mentioned were used with equal frequency.

**Table 2:** Forceps used and their use rates in our study.

Forceps	Use rates
Salinas	3.55%
Tucker-McLane	6.25%
Salas	11.00%
Kielland	32.50%
Simpson	46.70%

These are forceps available and their use at our hospital. Clearly, Simpson is the most favored due to its powerful traction, excellent results in prophylactic indications, and physician's familiarity with the instrument.
